# Plasmapheresis: Lifesaving treatment in severe cases of HELLP syndrome

**Published:** 2014

**Authors:** Jamshid Vafaeimanesh, Azam Nazari, Fatemeh Hosseinzadeh

**Affiliations:** 1 Clinical Research Development Center, Qom University of Medical Sciences, Qom, Iran.; 2 Shahid Beheshti Hospital, Qom University of Medical Sciences, Qom, Iran.

**Keywords:** HELLP syndrome, Plasmapheresis, Thrombocytopenia

## Abstract

***Background: ***HELLP syndrome is an important complications in pregnancy which increases maternal and fetal mortality. This disease usually remits with supportive treatment which includes prescription of corticosteroid, magnesium sulfate, stabilization of mother and pregnancy termination. Plasmapheresis is a treatment of choice which improves clinical outcomes in complicated cases.

***Case presentation:*** A 22-year-old woman with coagulopathy and respiratory distress and 33-year-old woman with a history of cerebellar medulloblastoma at 32-weeks’ gestation developed thrombocytopenia due to HELLP syndrome were treated with plasmapheresis were described.

***Conclusion: ***Plasmapheresis can significantly improve the outcome of patients with HELLP syndrome who are unresponsive to conservative therapy.

HELLP syndrome is a serious complication in pregnancy which was described as a severe form of preeclampsia in 1982 by Weinstein. HELLP stands for: H: hemolysis; EL: elevated liver enzymes; and LP: low platelets count. There is also partial or incomplete form of the disease which includes one or two parts of the triad (1). This syndrome was first assumed to be a severe form of preeclampsia but later it was revealed that it occurs in 10 to 20% of cases without preeclampsia (2). 

It is believed that HELLP syndrome affects about 0.5 to 0.9% of all pregnancies but its prevalence is higher in patients with preeclampsia and even can superimpose to 10-20% of patients with preeclampsia (3, 4). HELLP syndrome usually begins during the third trimester with the peak frequency between 27^th^ to 37^th^ gestational weeks; 30% occur after delivery (1, 4). It may also occur within 48 hours of delivery, the diagnosis of HELLP syndrome was confirmed after the development of dissemmated intravascular coagulation. 

Treatment of HELLP syndrome is primarily based on the gestational age but pregnancy termination is the treatment of choice to prevent any complication for the mother and the baby. In cases accompanied with disseminated intravascular coagulation, conservative treatment is forbidden (5). If the symptoms of HELLP begin to worsen after supportive treatment, delivery is the recommended course of treatment (1). It is important to take note that conservative treatment increases the rate of placental abruption, acute renal failure, pulmonary edema, disseminated intravascular coagulation and increasing risk of maternal or fetal death (6).

In cases that HELLP syndrome takes place after delivery, it is usually followed by support treatment and/or corticosteroid therapy (dexamethasone), but there is a disagreement about the effect of this method (7). One effective and life-saving method is plasmapheresis which decreases the maternal mortality rate from 23.1% to 0% (8). In this paper, we described two patients who were treated with plasmapheresis.

## Case presentation

**The first case: **The patient was a 22-year-old woman, G1 P1, at 40 weeks pregnant referred to the natural vaginal delivery ward. She had had +2 proteinuria during pregnancy and because of hypertension; methyldopa (250 mg each 8 hours) was prescribed. After delivery, she had abnormal vaginal bleeding and after coagulation and liver function tests (table 1), she was admitted in the ICU with diagnosis of complicated HELLP syndrome class 1 (table 2). She had fever (38.5^o^c) and respiratory distress and so she underwent ventilator respiration following tracheal intubation. Broad-spectrum antibiotics (vancomycin and imipenem) and corticosteroid (dexamethasone 12 mg each 12 hours) were initiated. The peripheral blood smear demonstraded 1% schizocyte. ADAMTS13 antigen level was 1.34 µg/mL (NL=0.6-1.6 µg/mL) and ADAMTS13 autoantibody level was 3 unit/mL ( neg: <12 , pos>15). 

The patient was qualified for plasmapheresis. After 13 plasmapheresis sessions, the patient became stable and was weaned from the ventilator. Eventually, after 21 sessions of plasmapheresis, she was discharged with good general condition.

**Table 1:** Results of the laboratory tests before and after plasmapheresis

**Laboratory data**	**Unit**	**Before plasmapheresis**		**After plasmapheresis**	
		**Patient 1**	**Patient 2**	**Patient 1**	**Patient 2**
Hemoglobin	g/dL	6	11.1	12	9.8
E-reticulocytes	%	4	2.5	1.5	1.3
Platelet count	**×**103/mm3	39	21	220	145
Urine analysis	-	+2	+3	-	-
Prothrombin time	Sec	22.9	12.2	13.2	12
INR	-	3	1	1.2	1
Partial Thromboplastin Time	Sec	43	25	25	24
Aspartate aminotransferase	IU/L	71	76	30	24
Alanine aminotransferase	IU/L	42	84	22	19
Bilirubin-total	mg/dL	5.9	1.7	1.3	0.9
Lactate dehydrogenase	IU/L	1120	1540	420	529
Creatinine	mg/dL	1.3	1.2	0.6	0.5
Number of plasmapheresis sessions				22	3

**Table 2:** The Mississippi-Triple Class System (1)

**HELLP class**	**Platelet**	**AST or ALT**	**LDH**
Class 1	≤50.10^9^/L	≥70 U/L	>600 U/L
Class 2	≤100. 10^9^/L ≥50. 10^9^/L	≥70 U/L	>600 U/L
Class 3	≤150. 10^9^/L ≥100. 10^9^/L	≥40 U/L	>600 U/L

**The second case: **A 35-year-old woman with a history of cerebellar medulloblastoma was hospitalized due to hypertension, monitored and was diagnosed with preeclampsia and due to gestational age, corticosteroid (dexamethasone 10 mg per 12 hours) was prescribed. Her blood pressure was controlled by intravenous hydralazine and methyldopa tablet on the third day of hospitalization, the platelets decreased to 20×10^9^/L and placental decolman occurred and the pregnancy was terminated immediately. The peripheral blood smear was observed and 2% fragmented RBC was reported. ADAMTS13 antigen level was 1.74 µg/mL (NL= 0.6-1.6 µg/mL) and ADAMTS13 autoantibody level was 5 unit/mL (neg: <12, pos>15).

Then, therapy with corticosteroid was preserved, but the thrombocytopenia persisted and finally on the third day following the delivery, the platelets reached 11×10^9^/L (table 1). Because of the continuation of thrombocytopenia despite the use of corticosteroid and dehydrogenase lactate 1530 IU/L, plasmapheresis was initiated. After 3 sessions, the patient was discharged in good general condition and normalized laboratory tests.

## Discussion

HELLP syndrome can cause several complications in fetus and mother and the mortality rate of these mothers is reported to be 1.1-25% (9, 10). Additionally, this complication can cause 7.4-4% of fetal deaths according to the time of onset (2, 11, 12). The most common cause of maternal death (directly or with fundamental role) is stroke or cerebral hemorrhage (13). Manrked elevation of liver enzyme may appear in some patients (14). 

In most women with HELLP syndrome, platelet count reduces after delivery. Three days after delivery, it reaches the lowest level (4). Sometimes HELLP syndrome occurs after delivery (1). In these cases, the usual treatment is 10 mg dexamethasone every 12 hours (4). The effectiveness of this treatment is not clearly defined and even some studies do not confirm it. On the contrary, some research demonstrated that plasmapheresis can significantly reduce the maternal mortality rate (8). Another noticeable point is the increase of coagulation and reduction of ADAMTS13 concentration in late pregnancy and postpartum period which increases the risk of thrombocytopenic thrombotic purpura in mothers. In some other cases, the differentiation between HELLP syndrome and thrombocytopenic thrombotic purpura is very difficult particularly when the measurement of ADAMTS13 is not accessible and mother does not have the history of preeclampsia (15-17). In such cases, not performing plasmapheresis will have adverse effects (1). It is also possible in some cases where it is required to distinguish between these diseases, the ratio of LDH/AST can be used and if the result is higher than 22.12, the probability of thrombotic thrombocytopenic purpura is higher than HELLP syndrome (18).

The first reported case of this study was suffering from class 1 HELLP syndrome and simultaneously had disseminated intravascular coagulation. Because of the coincidence of two diseases, 22 sessions of plasmapheresis were required. In patients with HELLP syndrome where plasmapheresis is performed, the range of platelets after 24, 48 and 72 hours will increase 2.2, 3.6 and 4.5 times, respectively and the amount of lactate dehydrogenase 48 hours after plasmapheresis will reach <1000 IU/L (19).

**Table 3:** the suggested cases for performing plasmapheresis in HELLP syndrome

**Suggested cases**	**Number of patients**	**Type of study**
Unusual HELLP syndrome or with low response to treatment (Progressive elevation of bilirubin or creatinine, constant thrombocytopenia (<30000) or LDH>1000IU/L for more than 72 hours after delivery (19)	7	Case series
Sever HELLP syndrome (Class 1 or 2) (8)	29	Research article
Central Nervous System involvement (16)	1	Case report
Accompany with Disseminated Intravascular Coagulation (21)	1	Case report
Accompany with dysfunction of several organs (22)	1	Case report
Accompany with cardiopulmonary complications (20)	1	Case report
When differentiation between HELLP syndrome and TTP is impossible (15)	-	Case series
When the disease is progressive during delivery or after that and in spite of using blood products (like bleeding) continues (23)	1	Case report

In spite of support treatment with corticosteroid within 3 days, the second case had the platelets 11.10^9^/L and lactate dehydrogenase 1530 IU/L; So, plasmaphersis was considered for treatment of these patients. After 3 sessions, the platelets and lactate dehydrogenase became normal. The reduction of total maternal mortality rate is another advantage of plasmapheresis. One study revealed that within 3 years, the mortality rate decreased from 23.1% to 0%. Also, the duration of admission in ICU will decrease and quick improvement in ALT, AST and LDH will occur (8).

The above statements reflect the importance of performing plasmapheresis in patients with HELLP syndrome which significantly improve the clinical outcomes and should be considered in such cases. The other research recommendations of this issue are shown in table 3. Also, in some studies, it has been pointed out that early plasmapheresis can improve the condition (8, 20). Therefore, it is suggested that physicians consider plasmapheresis in eligible cases of HELLP syndrome to improve the condition.
